# Secondhand Tobacco Smoke Exposure in Open and Semi-Open Settings: A Systematic Review

**DOI:** 10.1289/ehp.1205806

**Published:** 2013-05-07

**Authors:** Xisca Sureda, Esteve Fernández, María J. López, Manel Nebot

**Affiliations:** 1 Tobacco Control Unit, Cancer Control and Prevention Programme, Institut Català d’Oncologia-ICO, L’Hospitalet de Llobregat, Barcelona, Spain; 2 Cancer Control and Prevention Group, Institut d’Investigació Biomèdica de Bellvitge-IDIBELL, L’Hospitalet de Llobregat, Barcelona, Spain; 3 Department of Clinical Sciences, School of Medicine, Universitat de Barcelona, L’Hospitalet del Llobregat, Barcelona, Spain; 4 Agència de Salut Pública de Barcelona, Barcelona, Spain; 5 Institut d’Investigació Biomèdica-IBB Sant Pau, Barcelona, Spain; 6 Department of Experimental and Life Sciences, Universitat Pompeu Fabra, Barcelona, Spain

**Keywords:** exposure markers, outdoor tobacco smoke, particulate matter, passive smoking, secondhand smoke, smoking ban, tobacco smoke pollution

## Abstract

Background: Some countries have recently extended smoke-free policies to particular outdoor settings; however, there is controversy regarding whether this is scientifically and ethically justifiable.

Objectives: The objective of the present study was to review research on secondhand smoke (SHS) exposure in outdoor settings.

Data sources: We conducted different searches in PubMed for the period prior to September 2012. We checked the references of the identified papers, and conducted a similar search in Google Scholar.

Study selection: Our search terms included combinations of “secondhand smoke,” “environmental tobacco smoke,” “passive smoking” OR “tobacco smoke pollution” AND “outdoors” AND “PM” (particulate matter), “PM_2.5_” (PM with diameter ≤ 2.5 µm), “respirable suspended particles,” “particulate matter,” “nicotine,” “CO” (carbon monoxide), “cotinine,” “marker,” “biomarker” OR “airborne marker.” In total, 18 articles and reports met the inclusion criteria.

Results: Almost all studies used PM_2.5_ concentration as an SHS marker. Mean PM_2.5_ concentrations reported for outdoor smoking areas when smokers were present ranged from 8.32 to 124 µg/m^3^ at hospitality venues, and 4.60 to 17.80 µg/m^3^ at other locations. Mean PM_2.5_ concentrations in smoke-free indoor settings near outdoor smoking areas ranged from 4 to 120.51 µg/m^3^. SHS levels increased when smokers were present, and outdoor and indoor SHS levels were related. Most studies reported a positive association between SHS measures and smoker density, enclosure of outdoor locations, wind conditions, and proximity to smokers.

Conclusions: The available evidence indicates high SHS levels at some outdoor smoking areas and at adjacent smoke-free indoor areas. Further research and standardization of methodology is needed to determine whether smoke-free legislation should be extended to outdoor settings.

Secondhand smoke (SHS) is a complex mixture of thousands of compounds including particulate matter emitted by the combustion of tobacco products and from smoke exhaled by smokers [International Agency for Research on Cancer (IARC) 2004]. It contains > 50 chemicals recognized as known and probable human carcinogens, other animal carcinogens, and many toxic and irritant agents (U.S. Department of Health and Human Services 2006). Over the past two decades, scientific evidence has accumulated linking SHS exposure to adverse health outcomes, including respiratory outcomes in children and adults, acute cardiovascular effects, and lung cancer (IARC 2004; Ott et al. 2006; U.S. Department of Health and Human Services 2006). Most of this evidence is based on long-term SHS exposure research (IARC 2004). Some recent studies have also reported evidence of effects following short-term exposure to tobacco smoke, such as eye irritation and respiratory irritation among nonsmokers (Junker et al. 2001). Even brief and short-term exposures to SHS may generate significant adverse effects on the human respiratory system, as discussed in a recent review (Flouris and Koutedakis 2011). Finally, Pope et al. (2001) suggested that effects of acute exposure to tobacco smoke on cardiac autonomic function may contribute to pathophysiological mechanisms linking exposure to SHS to increased risk of cardiovascular mortality.

Smoke-free policies have been expanding worldwide since the World Health Organization (WHO) encouraged countries to follow Article 8 of the Framework Convention on Tobacco Control (FCTC) (WHO 2003) to protect people from SHS (Globalsmokefree Partnership 2009). Legislation has been widely implemented in indoor public places, workplaces, and public transportation (WHO 2009). Since the implementation of indoor smoke-free environments, several studies have demonstrated important reductions of SHS exposure, including an 80–90% decrease in previously high-exposure settings, such as workplaces and hospitality venues such as bars and restaurants (IARC 2008). However, indoor smoking bans may increase the likelihood that smokers will gather at convenient outdoor locations such as public areas near building entrances (Kaufman et al. 2010a). In 2007, a revision of the FCTC Article 8 guidelines further recommended that quasi-outdoor and outdoor public places should be smoke-free under some circumstances, and called upon countries to “adopt the most effective protection against exposure wherever the evidence shows that hazard exists” (WHO 2009). Recently, some countries have extended smoking bans to some outdoor locations (Globalsmokefree Partnership 2009; Repace 2008), particularly health care centers and settings where children are present (Globalsmokefree Partnership 2009). However, there remain some outdoor locations close to smoke-free areas where people may be exposed to SHS, such as terraces and patios in hospitality venues and near entrances to smoke-free buildings (Globalsmokefree Partnership 2009).

Some controversy exists regarding whether smoking should be prohibited in outdoor settings (Chapman 2008; Thomson et al. 2008). Health concerns about SHS exposure, nuisance from SHS, litter, fire hazards, concern about establishing positive smoke-free models for youth, and reducing youth opportunities to smoke (Bloch and Shopland 2000; Brennan et al. 2010; Cameron et al. 2010; Chapman 2008; Repace 2008; Thomson et al. 2008, 2009) exemplify the reasons why smoking should be banned in selected outdoor locations. Outdoor smoking bans might also support smokers who are trying to quit by limiting their overall cigarette consumption (Williams et al. 2009). Selected outdoor smoking bans should also help to denormalize smoking in outdoor areas (Thomson et al. 2008). In a number of jurisdictions, the majority of the public supports restricting smoking in various outdoors settings, and this support appears to be increasing over time (Thomson et al. 2009). However, those who oppose outdoor smoking bans argue that it is ethically unsustainable because it does not respect the principle of freedom and autonomy of individuals, and that there is insufficient evidence that SHS in these environments has an impact on health (Chapman 2000, 2008).

SHS exposure has been commonly studied in different indoor locations, especially in workplaces such as hospitality venues or health care centers (IARC 2009); however, outdoor SHS has been scarcely evaluated. It has been hypothesized that the introduction of indoor smoking bans has led to a relocation of smokers to outdoor areas, with a subsequent increase of tobacco smoke levels in outdoor places (Sureda et al. 2012). The aim of the present study is to review research on objectively assessed SHS levels in outdoor settings, including information on indoor and outdoor SHS concentrations, the effect of smoking bans on indoor and outdoor SHS levels, the relation between outdoor and indoor SHS levels, factors that influence outdoor and indoor SHS concentrations, and whether measured SHS levels comply with the air quality standards established by the WHO (2005).

## Methods

We conducted several different searches in PubMed (http://www.ncbi.nlm.nih.gov/pubmed) for papers published before September 2012 to identify papers on SHS assessment in outdoor settings. We combined different terms as follows:

((“Secondhand smoke” OR “environmental tobacco smoke” OR “passive smoking” AND “outdoor”) OR (“Tobacco Smoke Pollution”[Mesh] AND “outdoor”)) AND (PM OR RSP OR PM2.5 OR particulate matter OR nicotine OR CO OR cotinine OR marker OR markers OR biomarker OR airborne marker) AND (English[lang] OR French[lang] OR German[lang] OR Italian[lang] OR Spanish[lang] OR Catalan[lang]).

The search was more sensitive than specific; therefore, we arrived at the first selection of manuscripts by checking the results of every search and reading titles and abstracts. We then obtained the selected papers and read them carefully. Finally, we completed our search by checking the references of the papers and conducting similar searches in Google Scholar (http://www.scholar.google.com/; with search terms in English).

Our final selection included studies whose main objectives were to measure SHS or tobacco smoke exposure in outdoor settings using a tobacco biomarker or airborne marker. Outdoor areas included completely open spaces and quasi-outdoor areas with temporary or permanent structures, such as a roof or side walls, that would impede upward or lateral airflow, respectively.

We excluded articles that studied SHS exposure indoors but not outdoors and articles that studied air pollution outdoors, but not specifically SHS. We were able to consider papers in English, French, German, Italian, Spanish, and Catalan.

## Results

**Figure 1:**
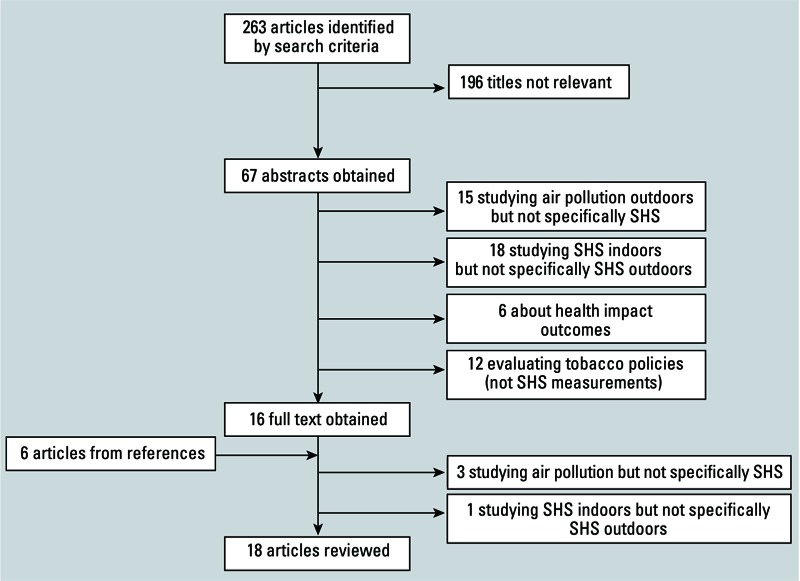
Flow diagram for the identification and selection of studies included in the review.

Our initial searches identified 263 papers; after checking the titles, 67 abstracts were reviewed (Figure 1). Of these, 51 were determined not to meet eligibility criteria. We read the remaining 16 papers in full, plus 6 additional papers identified from references. We finally identified 18 articles and reports that satisfied the inclusion criteria, including 15 published in peer-review journals and 3 academic reports available on the Internet. One report was a pilot study for which we obtained data from the subsequently published study (Klepeis et al. 2007). We included only results related to SHS in outdoor areas from another report [California Air Resources Board (CARB) 2005] concerning SHS exposure in California.

The 18 papers included were published between 2005 and 2012. The studies were conducted in Australia (*n* = 3), Canada (*n* = 2), New Zealand (*n* = 4), the United States (*n* = 6), Denmark (*n* = 1), and Spain (*n* = 1), and a multicenter study was conducted in eight European countries (*n* = 1) (Table 1). Almost all (*n* = 16) used airborne markers to assess SHS exposure, including 14 studies that measured particulate matter ≤ 2.5 µm in diameter (PM_2.5_). Airborne nicotine, carbon monoxide (CO), PM_3.5_ (≤ 3.5 µm in diameter), and polycyclic aromatic hydrocarbons (PAHs) were used infrequently and mostly to complement PM_2.5_ assessment (*n* = 5). Two studies used personal biological markers {salivary cotinine in both studies and NNAL [4-(methylnitrosamino)-1-(3-pyridyl)-1-butanol] in one of the studies} to assess tobacco exposure among participants (Hall et al. 2009; St.Helen et al. 2012).

**Table 1:** Main characteristics of reviewed studies from before September 2012 assessing outdoor SHS exposure in hospitality venues.

Reference, location	Study design: venue type, and sample size	SHS marker	Potential confounders	SHS marker concentration		Background concentration (control)
				Presence of smokers	Absence of smokers	
Klepleis etal. 2007, California, USA	Observational and experimental: 10 outdoor public places including parks, sidewalk cafés, and restaurant and pub patios. Results provided for hospitality venues and other settings combined	PM_2.5_	Wind conditions, source proximity, and no. of cigarettes	Overallmean:30µg/m^3^ (observational data). Maximum: 1,000µg/m^3^ at distances within 0.5 m (experimental data)
Travers etal. 2007, Victoria, British Columbia, Canada	Observational: 20 smoking areas of bars and restaurants (outdoors)	PM_2.5_	No. of burning cigarettes, coverage and cigarette proximity, or size	Overall mean: 96µg/m^3^. Maximum: 1,318µg/m^3^		6µg/m^3^
Wilson etal. 2007, New Zealand	Observational: 34 pubs, restaurants, and bars; 6 outdoor smoking areas of bars and restaurants. Also in this study: 10transportation settings, 9other indoor settings, and 6 other outdoor settings (Table2)	PM_2.5_	No. of people in room/area and no. of lit cigarettes among occupants	“Outdoor” smoking areas of bars and restaurants (*n*=4): 36µg/m^3^. Relatively enclosed smoking areas attached to bars (*n*=2): 124µg/m^3^. Maximum (outdoor smoking area in a bar): 284µg/m^3^	Inside hospitality venues (*n*=34): 16µg/m^3^. Outside hospitality venues (*n*=34): 14µg/m^3^	14µg/m^3^
Hall etal. 2009, Athens, Georgia, USA	Observational: 5 bars (*n*=3) and family restaurants (*n*=2) (outdoors)	SC	Proximity to smokers	Overall GM, bar: 182µg/m^3^. Overall GM, restaurant: 75µg/m^3^	Overall GM, bar: 69µg/m^3^. Overall GM,restaurant: 36µg/m^3^	Before smoking time: 43µg/m^3^. After smoking time: 49µg/m^3^
Brennan etal. 2010, Victoria, Australia	Observational: 19 pubs and bars that had at least one indoor area with an adjacent semi-enclosed outdoor eating/drinking area (5m from the main access)	PM_2.5_	No. of patrons and lit cigarettes, overhead covers, ventilation, and kitchen operating	OverallGMindoor: 61.3µg/m^3^ (pre-ban). OverallGM, outdoor: 19.0µg/m^3^ (pre-ban)	Overall GM, indoor: 17.4µg/m^3^ (post-ban). Overall GM, outdoor: 13.1µg/m^3^ (post-ban)	
Cameron etal. 2010, Melbourne, Australia	Observational: 69 visits to 54 dining areas of bars and restaurants	PM_2.5_	No. of target cigarettes, no. of other lit cigarettes, and overhead cover	Overall mean: 27.3µg/m^3^. Maximum: 483.9µg/m^3^	Overall mean: 17.6µg/m^3^	8.4µg/m^3^
Stafford etal. 2010, Perth and Mandurah, Australia	Observational: 12 cafes and 16pubs (outdoors)	PM_2.5_	No. of smokers, wind level, coverage, no. of patrons, street type, and road traffic	Overall median: 8.32µg/m^3^. Maximum: 142.08µg/m^3^	Overall median: 2.56µg/m^3^
Edwards etal. 2011, New Zealand	Observational: 7 pubs and bars (semi-enclosed outdoor area and indoor)	PM_2.5_	Ventilation	Noncommunication smoking area outdoors: range, 32–109µg/m^3^. Communication smoking area outdoors: range, 29–192µg/m^3^	Noncommunication smoking area indoors: range, 14–79µg/m^3^. Communication smoking area indoors: range, 2.36–117µg/m^3^
St.Helen etal. 2011, Athens, Georgia, USA	Observational: 2 family restaurants, 3 bars (outdoors)	PM_2.5 _and CO	No. of smokers, pedestrians, and vehicles	PM_2.5_: range, 16.6–63.9µg/m^3^. CO: range, 1.2–1.6 ppm		PM_2.5_: 20.4µg/m^3^. CO: 1.3 ppm
Wilson etal. 2011, New Zealand	Observational: 20 outdoor smoking areas of hospitality venues, 13inside bars adjacent to outdoor smoking areas, 10 pubs/sports bars, 18 bars, 9restaurants, 5cafés. Also in this study: 15inside public buildings, 15inside transportation settings, and 22 various outdoor street/park settings	PM_2.5_	None	Outdoor smoking areas of hospitality venues (*n*=20): 72µg/m^3^. Inside bars adjacent to outdoor smoking areas (*n*=13): 54µg/m^3^	Inside hospitality venues (*n*=42): range, 7–22µg/m^3^	11µg/m^3^
St.Helen etal. 2012, Athens, Georgia, USA	Observational: a bar and a family restaurant (outdoors), an open-air seating area with no smokers (control)	SC and NNAL	No. of lit cigarettes	SC in restaurant: 69µg/m^3^. SC in bar: 165µg/m^3^. NNAL, in restaurant: 0.774µg/m^3^. NNAL in bar: 2.407µg/m^3^	SC in restaurant: 46µg/m^3^. SC in bar: 45µg/m^3^. NNAL in restaurant: 0.041µg/m^3^. NNAL in bar: 0.037µg/m^3^	SC: 53µg/m^3^. NNAL: 0.038µg/m^3^
López etal. 2012, Europe	Observational: 48 hospitality venues (night bars, restaurants and bars)	PM_2.5_ and nicotine	No. of smokers and coverage	PM_2.5_ indoors (*n*=42): 120.51µg/m^3^ (pre-ban). PM_2.5_ outdoors (*n*=42): 29.61µg/m^3^ (pre-ban). Nicotine indoors (*n*=46): 3.69µg/m^3^ (pre-ban). Nicotine outdoors (46): 0.31µg/m^3^ (pre-ban)	PM_2.5_ indoors (32): 36.90µg/m^3^ (post-ban). PM_2.5_ outdoors (32): 36.10µg/m^3^ (post-ban). Nicotine indoors (39): 0.48µg/m^3^ (post-ban). Nicotine outdoors (39): 1.56µg/m^3^ (post-ban)
Abbreviations: GM, geometric mean; NNAL, 4-(methylnitrosamino)-1-(3-pyridyl)-1-butanol; SC, salivary cotinine.						

The studies included between 2 and 127 locations. Depending on the specific study objectives, different locations were tested. Nine studies were conducted in hospitality venues (Table 1) such as pubs, restaurants, bars, cafés, and outdoor dining areas. Six studies measured SHS in other locations such as entrances to buildings and the adjacent indoor area and transportation settings, including an airport, parks, streets, university campuses, and one junior college campus (Table 2). Three studies assessed SHS in both hospitality and non-hospitality venues. Most studies were observational studies, with only two experimental studies. All included papers were written in English.

**Table 2:** Main characteristics of reviewed studies from before September 2012 assessing outdoor SHS exposure in non-hospitality settings.

Reference, location	Study design: venue type, and sample size	SHS marker	Potential confounders	SHS marker concentration		Background concentration (control)
				Presenceofsmokers	Absenceofsmokers	
CARB 2005, California, USA	Observational: an airport, a junior college campus, a public building, an office complex, and a park	Airborne nicotine	No. of cigarettes smoked, wind speed, and direction	Range, 0.013–3.1µg/m^3^		Range, 0.009–0.12µg/m^3^
Repace 2005, Baltimore, USA	Experimental: various locations on the UMBC campus (outdoors and indoors)	PM_3.5_ and PAH	Distances, number of smokers, and wind conditions	Range, 100–150µg/m^3^ outdoors in proximity to smokers
Boffi etal. 2006, Copenhagen, Denmark	Observational: in a car park, inside a nonsmoking conference center, outdoors in front of the conference center, with smokers under a roof, along the motorway, and inside a Copenhagen restaurant where smoking was allowed	PM_2.5_	None	Outside in front of a conference center: 17.8µg/m^3^. Along the motorway: 4.6µg/m^3^	Car parking area: 6.0µg/m^3^. Inside a conference center: 3.0µg/m^3^	5.7µg/m^3^
Klepeis etal. 2007, California, USA	Observational and experimental: 10 outdoor public places including parks, sidewalk cafés, and restaurant and pub patios. Results provided for hospitality venues and other settings combined	PM_2.5_	Wind conditions, source proximity, and no. of cigarettes	Overall mean: 30µg/m^3^. Maximum: 1,000µg/m^3^ at distances within 0.5 m
Wilson etal. 2007, New Zealand	Observational: 10 transportation settings, 9 non-hospitality indoor settings, and 6 non-hospitality outdoor settings. Also in this study: 34 pubs, restaurants, and bars and 6outdoor smoking areas of bars and restaurants	PM_2.5_	No. of people in room/area and no. of lit cigarettes among occupants		Transportations settings (*n*=10): 13µg/m^3^. Non-hospitality indoors (*n*=9): 3µg/m^3^. Non-hospitality outdoors (*n*=6): 7µg/m^3^	14µg/m^3^
Kaufman etal. 2010b, Toronto, Canada	Observational: entrances to 28 office buildings both indoor and outdoor	PM_2.5_	No. of cigarettes, wind direction and strength, and distance from the nearest lit cigarette to the monitor	Overall median outdoors: 11µg/m^3^ (1–4 cig); 16µg/m^3^ (≥5 cig). Maximum: 496µg/m^3^. Overall median indoors: 6µg/m^3^ (1–4 cig); 4µg/m^3^ (≥5 cig)	Overall median outdoors: 8µg/m^3^. Overall median indoors: 5µg/m^3^	8µg/m^3^
Parry etal. 2011, New Zealand	Observational: streets (no. of samples not indicated)	PM_2.5_	No. of smokers, smoking proximity, and coverage	Overall mean: 14.2µg/m^3^. Maximum: 186.0µg/m^3^	Overall mean: 5.9µg/m^3^
Sureda etal. 2012, Barcelona, Spain	Observational: 47 public building main entrances (both outdoors and indoors)	PM_2.5_ and airborne nicotine	No. of lit cigarettes, coverage, and distance to roadways	Overall PM_2.5_ concentration outdoor: 17.16µg/m^3^. Overall PM_2.5_ concentration indoor: 18.20µg/m^3^. Nicotine concentration in 28main entrances outdoors: 0.81µg/m^3^. Maximum value PM_2.5_ (outdoor): 128.44µg/m^3^	Overall PM_2.5_ concentration Control point indoor: 10.40µg/m^3^	PM_2.5_ concentration: 13.00µg/m^3^
Wilson etal. 2011, New Zealand	Observational: 15 inside public buildings, 15 inside transportation settings, and 22 various outdoor street/park settings. Also in this study: 20 outdoor smoking areas of hospitality venues, 13 inside bars adjacent to outdoor smoking areas, 10 pubs/sports bars, 18 bars, 9restaurants, and 5 cafés	PM_2.5_	None		Inside non-hospitality settings (*n*=30): range, 2–13µg/m^3^. Non-hospitality outdoor settings: range, 2–11µg/m^3^	11µg/m^3^
cig, cigarettes.						

**Figure 2:**
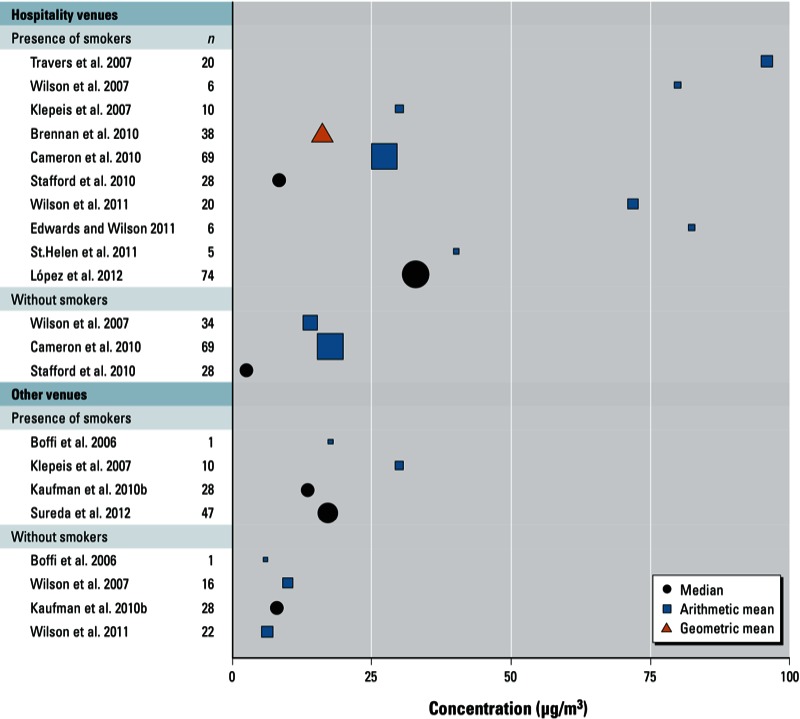
Outdoor PM_2.5_ concentrations reported for hospitality venues and other settings according to the presence or absence of smokers. Klepleis et al. (2007) included hospitality and non-hospitality venues without distinguishing the mean value between them, and hence it has been included both in “hospitality venues” and “other venues.” Wilson et al. (2011) and Edwards and Wilson (2011) provided the individual values for each measurement, and we have computed the arithmetic mean for the figure. Brennan et al. (2010) and López et al. (2012) provided mean and median values, respectively, for venues before and after a smoking ban. We have computed the average values for each study to include them in the figure.

*SHS in outdoor smoking areas*. Mean PM_2.5_ concentrations reported for outdoor smoking areas at hospitality venues ranged from 8.32 µg/m^3^ (Stafford et al. 2010) to 124 µg/m^3^ (Wilson et al. 2007) when smokers were present (Table 2). In non-hospitality venues, mean PM_2.5_ concentrations reported for outdoor settings ranged from 4.60 µg/m^3^ (Boffi et al. 2006) to 17.80 µg/m^3^ (Boffi et al. 2006) (Figure 2). Klepeis et al. (2007) obtained an overall PM_2.5_ mean of 30 µg/m^3^ for the observational data for hospitality venues and other settings combined. In the experimental component of the same study, PM_2.5_ concentrations reached values of 200 µg/m^3^ and 500 µg/m^3^ depending on other external conditions (Klepeis et al. 2007).

Three studies (Cameron et al. 2010; Parry et al. 2011; Stafford et al. 2010) that compared outdoor SHS measurements during smoking and nonsmoking periods reported that particulate concentrations were significantly higher during active smoking. Two studies reported that PM_2.5_ concentrations in outdoor smoking areas were higher than background PM_2.5_ levels similarly measured in nearby, smoke-free, outdoor air (St.Helen et al. 2011; Travers et al. 2007). An additional study (Boffi et al. 2006) reported high PM_2.5_ concentrations both outdoors and indoors during 1 day in a conference center where smoking was permitted.

One study used salivary cotinine to evaluate SHS exposures among nonsmokers before and after they spent 6 hr at smoking areas of outdoor bars or outdoor restaurants, or at an outdoor control site without smoking (Hall et al. 2009). Median increases in salivary cotinine from pretest to posttest were approximately 162%, 102%, and 16% for the bar, restaurant, and control sites, respectively. A similar study measured salivary cotinine in saliva and NNAL in urine samples from non-smokers before and after being at an outside bar or restaurant or at a control site (St.Helen et al. 2012). Cotinine in samples collected both immediately after and the morning after 3-hr visits to the outside bar and restaurant sites were significantly higher than in the control samples, and NNAL was significantly higher in first morning urine samples after bar and restaurant site visits. Another study used airborne nicotine to assess SHS exposure; the mean 8-hr concentrations ranged from 0.013 to 3.1 µg/m^3^ (higher than the mean 8-hr background concentrations of 0.009–0.12 µg/m^3^) (CARB 2005).

*Factors influencing outdoor SHS levels*. Atmospheric conditions, including wind direction, wind speed, and atmospheric stability, can modify outdoor SHS levels. Other factors are the density and distribution of the smokers and the structure of the outdoor location (completely open or semi-open). All of the studies that evaluated possible modifiers of SHS concentrations reported that the density of smokers and/or number of lit cigarettes predicted outdoor SHS (Brennan et al. 2010; Cameron et al. 2010; CARB 2005; Edwards and Wilson 2011; Kaufman et al. 2010b; Klepeis et al. 2007; López et al. 2012; Parry et al. 2011; Repace 2005; St.Helen et al. 2011, 2012; Stafford et al. 2010; Sureda et al. 2012). Most of these studies also found the degree of enclosure of the outdoor area as a determinant factor (Brennan et al. 2010; Cameron et al. 2010; López et al. 2012; Parry et al. 2011; Stafford et al. 2010; Sureda et al. 2012; Travers et al. 2007). For example, Cameron et al. (2010) reported that PM_2.5_ increased by approximately 30% with each additional active smoker within 1 m of the point of measurement, and by 50% if measured under an overhead cover.

Some studies on wind conditions (speed and direction) and proximity to smokers found that these were not associated with SHS levels (Kaufman et al. 2010b; Travers et al. 2007). However, the CARB study (2005) and two experimental studies (Klepeis et al. 2007; Repace 2005) in public outdoor locations that controlled smoking activity at precise distances from monitored positions reported that outdoor SHS levels were highly dependent on wind direction and source proximity. Klepeis et al. (2007) demonstrated that upwind PM_2.5_ concentrations are likely to be very low, whereas downwind levels during periods of active smoking can be very high. They also reported that PM_2.5_ levels decreased by half or more as the distance from a lit cigarette increased from 0.25–0.5 m to 1–2 m, and that levels were generally close to background. However, Repace (2005) reported that outdoor PM_3.5_ and PAH concentrations did not approach background levels until about 7 m.

*Outdoor smoking areas and indoor air quality*. PM_2.5_ concentrations in indoor settings where smoking was banned but near outdoor smoking areas varied from 4 µg/m^3^ (Kaufman et al. 2010b) to 120.51 µg/m^3^ (López et al. 2012); both studies were carried out in hospitality venues. Indoor PM_2.5_ levels far away from outdoor tobacco sources were lower (Sureda et al. 2012; Wilson et al. 2011).

Two studies specifically examined SHS in main entrances of public buildings. Kaufman et al. (2010b) simultaneously measured PM_2.5_ concentrations inside and outside of 28 office building entrances. Outdoor SHS levels within 9 m of building entrances were significantly higher in the presence of smoking (11 µg/m^3^ with 1–4 cigarettes, and 16 µg/m^3^ with ≥ 5 cigarettes) compared to occasions when there was no smoking (8 µg/m^3^). PM_2.5_ median indoor concentrations ranged from 4 to 6 µg/m^3^. Sureda et al. (2012) showed higher median PM_2.5_ concentrations in the presence of smoking, both outdoors near main entrances (17.16 µg/m^3^) and in indoor halls near outdoor smoking areas (18.20 µg/m^3^), compared with those in control locations without smoking, both indoors (10.40 µg/m^3^) and outdoors (13.00 µg/m^3^).

Several articles reported positive associations between SHS levels (PM_2.5_ concentrations) measured indoors and outdoors (Brennan et al. 2010; Edwards and Wilson 2011; Kaufman et al. 2010b; López et al. 2012; Sureda et al. 2012; Wilson et al. 2011). Indoor SHS levels are higher when smoking occurs in the adjacent outdoor setting, especially when the outdoor area is semi-enclosed. For example, Sureda et al. (2012) showed that PM_2.5_ concentrations in indoor halls were more closely correlated with outdoor concentrations measured near main entrances (outdoors) than with the indoor control (a nonsmoking area far from the main entrance). Brennan et al. (2010) estimated that a 100% increase in the geometric mean of the outdoor PM_2.5_ concentration was associated with a 36.1% rise in the geometric mean of the indoor PM_2.5_ concentration in smoke-free pubs and bars.

*Factors influencing indoor SHS from outdoor areas*. Factors such as wind speed and direction that modify outdoor SHS levels also may influence indoor air quality. The effects of structural barriers between outdoor smoking areas and indoor locations were also considered in some articles (Brennan et al. 2010; Edwards and Wilson 2011). Brennan et al. (2010) observed that open access between indoors and outdoors was associated with lower PM_2.5_ levels indoors. However, an Australian study (Edwards and Wilson 2011) showed higher indoor PM_2.5_ concentrations when doors to outdoor smoking areas were left open.

*Smoking bans and SHS exposures*. One study evaluated the impact of laws prohibiting indoor smoking (Brennan et al. 2010) by measuring PM_2.5_ concentrations before and after indoor smoking bans were implemented in pubs and bars that had at least one indoor area with an adjacent semi-enclosed outdoor eating/drinking area, and showed reduced PM_2.5_ concentrations both indoors and outdoors (65.5% and 38.8%, respectively) from pre-ban to post-ban. Two other studies evaluated indoor and outdoor SHS in different settings after the implementation of indoor smoking bans (Wilson et al. 2007, 2011). Both reported higher concentrations of fine particulates in outdoor smoking areas, especially those that were partly enclosed, as well as indoor areas adjacent to outdoor smoking areas compared to other smoke-free indoor settings. Finally, a multicenter study carried out in hospitality venues of eight European countries compared SHS concentrations between venues where indoor smoking was allowed and venues where it was banned (López et al. 2012). The authors reported that median indoor PM_2.5_ and airborne nicotine concentrations were significantly higher in venues where smoking was allowed than in those where it was banned. Conversely, the outdoor nicotine concentration was significantly higher for venues where indoor smoking was banned than outdoor areas of venues where indoor smoking was allowed (López et al. 2012).

*Tobacco smoke levels compared to background levels*. Maximum mean or median outdoor PM_2.5_ concentrations ranged from 128 µg/m^3^ (Sureda et al. 2012) to 496 µg/m^3^ (Kaufman et al. 2010b), with some point measurements exceeding 1,000 µg/m^3^ (Klepeis et al. 2007; Travers et al. 2007). The maximum peak indoor PM_2.5_ concentration reported for a smoke-free setting was 239 µg/m^3^ (Wilson et al. 2011). In contrast, mean or median background PM_2.5_ concentrations varied from 6 µg/m^3^ (Travers et al. 2007) to 20.4 µg/m^3^ (St.Helen et al. 2011).

*SHS markers other than PM_2.5_*. Three studies evaluated different SHS markers to determine which would be most appropriate to describe SHS levels in outdoor areas. Sureda et al. (2012) reported a Spearman correlation coefficient between outdoor PM_2.5_ and airborne nicotine concentrations of 0.365 (95% CI: 0.009, 0.650). Hall et al. (2009) reported that the number of smokers present had a strong positive association with outdoor PM_2.5_ concentrations but not CO concentrations. Moreover, CO levels measured outside restaurants and bars did not differ significantly from concentrations measured at a control location, in contrast with findings for PM_2.5_ concentrations. Other studies used biological markers such as cotinine or NNAL to show SHS exposure (Hall et al. 2009; St.Helen et al. 2012).

## Discussion

We found only 18 studies that met our criteria, but these indicated that SHS levels in some outdoor smoking areas are not negligible, especially in areas that are semi-enclosed.

*SHS levels and air quality standards*. In general, SHS levels measured in outdoor smoking areas were high, particularly in hospitality venues where PM_2.5_ concentrations ranged from 8.32 µg/m^3^ (Stafford et al. 2010) to 182 µg/m^3^ (Hall et al. 2009) when smokers were present. SHS levels were also increased in indoor areas adjacent to outdoor smoking areas. Hall et al. (2009) and St.Helen et al. (2012) reported that saliva cotinine concentrations were higher in study participants following exposure to SHS at outdoor bars and restaurants when smoking was allowed than after exposure to smoke-free terraces. These results suggest that hospitality workers and patrons may be exposed to high SHS levels under certain conditions. Although outdoor SHS levels are more transient than indoor levels, and can quickly drop to background levels in the absence of active smoking, potential health effects of these exposures merit consideration and need to be further studied.

According to the WHO, there is no safe level of SHS (WHO 2000). The WHO guidelines indicate that the lower range of concentrations at which adverse health effects have been demonstrated is not greatly above background concentrations (estimated at 3–5 μg/m^3^ in the United States and Western Europe for PM_2.5_). In the updated WHO Air Quality Guidelines, an annual outdoor average value of 10 μg/m^3^ for PM_2.5_ was selected as the lower end of the range over which significant effects on survival have been observed (Gorini et al. 2005; WHO 2000, 2005). These are the lowest levels at which total, cardiopulmonary, and lung cancer mortality have been shown to increase with more than 95% confidence in response to PM_2.5_. Most of the reviewed studies of PM_2.5_ concentrations in outdoor smoking areas reported levels higher than the annual mean guideline value of 10 μg/m^3^ recommended by WHO

*Influences of outdoor SHS on indoor air quality*. Indoor smoke-free areas near outdoor smoking areas showed higher levels than smoke-free indoor areas that were farther away from outdoor SHS sources, suggesting that SHS from outdoor smoking areas can enter adjacent buildings. Some findings also suggested that although outdoor SHS concentrations dropped immediately to background levels when the SHS sources were extinguished, indoor SHS concentrations persisted at relatively high levels and slowly decayed over several hours until doors were opened to ventilate the building (Klepeis et al. 2007). SHS levels in outdoor locations are more susceptible to variation due to the proximity of active smoking and wind conditions. During periods of active smoking, outdoor SHS levels can be comparable to levels in indoor smoking areas, but outdoor levels dropped rapidly after smoking activity ceased.

*Other factors influence SHS levels*. Some factors can influence SHS levels both indoors and outdoors (Brennan et al. 2010; Cameron et al. 2010; Edwards and Wilson 2011; Kaufman et al. 2010b; Klepeis et al. 2007; López et al. 2012; Repace 2005; St.Helen et al. 2011, 2012; Stafford et al. 2010; Sureda et al. 2012). Smoker density and enclosure of the outdoor locations are determinant modifiers. Some studies also suggest that wind speed and direction, as well as proximity to smokers, are associated with SHS levels outdoors.

*SHS airborne markers other than PM_2.5_*. Particulate matter was the most common airborne marker used in the presently reviewed articles. However, PM_2.5_ is not a specific marker; markers such as airborne nicotine are specific to SHS (Gorini et al. 2005; Ott et al. 2006). Biological markers have been scantily used. However, cotinine has been proposed as a very sensitive and specific biological marker of SHS exposure (Benowitz 1999), and total NNAL has been used to characterize human exposure to carcinogenic tobacco-specific nitrosamines among nonsmokers exposed to SHS (Anderson et al. 2001). Further research is necessary to evaluate which SHS marker would be most appropriate to measure SHS levels in outdoors settings and whether it would be necessary to combine more than one marker.

*Limitations*. Some of the reviewed studies did not control for important factors that can influence SHS levels, such as wind conditions, the structural characteristics of outdoor area (semi-enclosed vs. totally open), or proximity to active smokers. Future studies should control for these factors to enable a better understanding of the results. Additionally, some studies used PM_2.5_ concentrations to estimate SHS levels in outdoor areas, but did not control for other sources of PM_2.5_, such as cooking or traffic-related air pollution (Gorini et al. 2005). Further studies should record the presence of other sources of combustion, such as cooking facilities, proximity to roadways, or traffic density; measure and report background levels of PM_2.5_; and/or use specific SHS markers such as airborne nicotine.

Publication bias is a potential source of error in systematic reviews. We searched the available literature in PubMed, the main biomedical database, and Google Scholar and checked references to identify documents not published in academic journals. However, we cannot rule out the possibility that some unpublished manuscripts or other documents addressing the topic of interest may have been missed. Direct comparisons of results among studies were hampered by the use of different statistics (medians, means, or geometric means) and sampling strategies; the use of standardized methods could strengthen the validity of results and facilitate comparisons among different populations and locations. Furthermore, the number of venues measured in each study was limited. Future studies should consider including representative samples of locations selected using standard statistical sampling procedures and sample size computations.

*Strengths*. The reviewed studies included a variety of venue types (e.g., entrances to public buildings, hospitality venues, transportation settings) and characteristics. Most of the reviewed studies were observational, and thus provide information that reflects smoking behaviors and exposures under normal real-life conditions. However, experimental studies provide the opportunity to control for unpredictable variables, such as the proximity of smokers or wind conditions. The use of real-time monitoring permits determination of the precise magnitude of extremely transient (short-term) concentrations and exposures, while retaining the flexibility of exploring concentrations and exposure across a variety of averaging times and time series and calculating mean concentrations and exposures (Klepeis et al. 2007).

## Conclusion

Only limited evidence is available regarding SHS exposure in outdoor settings as determined by environmental and biological markers; therefore, the existing evidence must be interpreted carefully. However, our review clearly indicates the potential for high SHS exposures at some outdoor settings and indoor locations adjacent to outdoor smoking areas. This review shows that high smoker density, highly enclosed outdoor areas, low wind conditions, and close proximity to smokers generate higher outdoor SHS concentrations. Accounting for these factors is important for future studies on the relationship between outdoor SHS exposure and health outcomes.

The WHO Framework Convention on Tobacco Control has concluded that 100% smoke-free environments are required to adequately protect the public’s health from the harmful effects of SHS (WHO 2003). The present review indicates that further research using standardized methodology is needed to better characterize outdoor SHS exposure levels and determine whether smoke-free legislation should be extended to outdoor areas.

Future studies should include representative samples of different locations; use standardized statistical analyses and report multiple measures of central tendency and measures of variability (standard errors, confidence intervals, or quartiles); and consider potential modifiers of SHS levels including smoker density, degree of enclosurement of outdoor locations, wind speed and direction, and proximity to smokers. Finally, further research is needed to determine the most appropriate marker or combination of markers to assess SHS exposure, which may include more specific environmental and individual markers of exposure (e.g., airborne nicotine and cotinine in saliva) in addition to PM_2.5_ concentration.
